# NMDA Receptors in Neurodevelopmental Disorders: Pathophysiology and Disease Models

**DOI:** 10.3390/ijms252212366

**Published:** 2024-11-18

**Authors:** Roshan Tumdam, Yara Hussein, Tali Garin-Shkolnik, Shani Stern

**Affiliations:** 1 Sagol Department of Neurobiology, Faculty of Natural Sciences, University of Haifa, Haifa 3103301, Israel; 2 Center for Rare Diseases, Emek Medical Center, Afula 1834111, Israel

**Keywords:** NDD—neurodevelopmental disorder, EPSCs—excitatory postsynaptic currents, mEPSCs—miniature excitatory postsynaptic currents, ATD—amino-terminal domain, LBD—ligand-binding domain, TMD—transmembrane domain, CTD—C-terminal domain, CNS—central nervous system, SCZ—schizophrenia, ASD—autism spectrum disorder

## Abstract

N-methyl-D-aspartate receptors (NMDARs) are critical components of the mammalian central nervous system, involved in synaptic transmission, plasticity, and neurodevelopment. This review focuses on the structural and functional characteristics of NMDARs, with a particular emphasis on the GRIN2 subunits (GluN2A-D). The diversity of GRIN2 subunits, driven by alternative splicing and genetic variants, significantly impacts receptor function, synaptic localization, and disease manifestation. The temporal and spatial expression of these subunits is essential for typical neural development, with each subunit supporting distinct phases of synaptic formation and plasticity. Disruptions in their developmental regulation are linked to neurodevelopmental disorders, underscoring the importance of understanding these dynamics in NDD pathophysiology. We explore the physiological properties and developmental regulation of these subunits, highlighting their roles in the pathophysiology of various NDDs, including ASD, epilepsy, and schizophrenia. By reviewing current knowledge and experimental models, including mouse models and human-induced pluripotent stem cells (hiPSCs), this article aims to elucidate different approaches through which the intricacies of NMDAR dysfunction in NDDs are currently being explored. The comprehensive understanding of NMDAR subunit composition and their mutations provides a foundation for developing targeted therapeutic strategies to address these complex disorders.

## 1. Introduction

**Figure 1:**
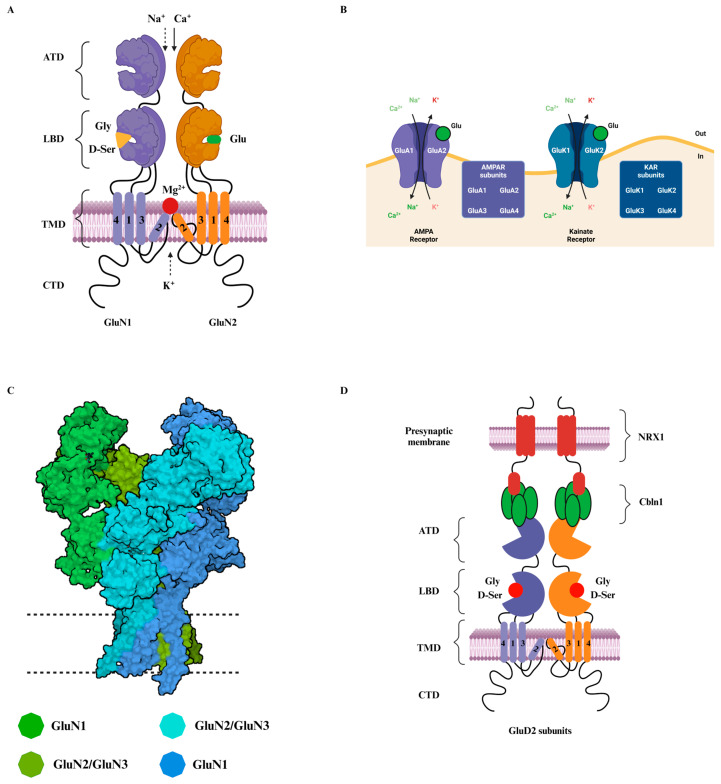
Structural composition of the NMDA receptors; (**A**) Graphical representation of the subunit composition of NMDA receptors. Each monomer of the receptor contains four functional domains, the amino-terminal domain (ATD), ligand-binding domain (LBD), transmembrane domain (TMD), and C-terminal domain. (**B**) Ion channel activity of AMPA and Kainate receptors (members of iGluR family). (**C**) Crystal structure displaying 3D conformation of the heterotetramer NMDA receptor containing GluN1 and GluN2B subunits (PDB: 6WHX); GluN3 subunits also form functional receptors with GluN1 subunit. (**D**) The proposed GluD2 ion channel activity mechanism is through interaction with pre-synaptic linker proteins; the representation is adapted from Carillo et al. SciAdv, 2021 [12].

The non-essential amino acid glutamate is the most dominant excitatory neurotransmitter in the CNS. It acts as a fast transmitter and plays a crucial role in modulating cellular excitability and synaptic transmission between neuronal networks [1]. Glutamate receptors (GluRs) are distributed widely across the mammalian brain, serving as the principal excitatory transmitter system. Based on the preference of these receptors towards different excitatory small molecule agonists, initially, the glutamate receptors were thought to be divided into NMDA and non-NMDA receptors, which were considered to mediate fast postsynaptic potentials by activating the ion channels directly [2]. Following the successful expression and functional studies of a glutamate receptor in Xenopus oocyte [3], a large family of glutamate receptor subunits was discovered, and a strong correlation between molecular subtype and pharmacological properties was established among the receptor subunits [4,5]. Based on the structural and functional aspects, GluRs in the CNS are majorly categorized into two families namely, metabotropic glutamate receptors (mGluRs), and ionotropic glutamate receptors (iGluRs) [6,7]. The mGluRs are a family of G-protein coupled receptors (GPCRs) that are activated by extracellular ligands such as neurotransmitters and mediate downstream signaling through the G-proteins [8,9]. The most classical neurotransmitter GPCRs are part of family A. mGluRs are class-C GPCRs with a unique structure characterized by a large extracellular N-terminal domain that houses the endogenous ligand-binding site [10]. Ionotropic glutamate receptors, on the other hand, are cation-permeable ligand-gated ion channels, including α-amino-3-hydroxy-5-methyl-4-isoxazolepropionic acid receptors (AMPARs), Kainate receptors (KARs), N-methyl-D-aspartate receptors (NMDARs), and delta receptors, also known as orphan receptors (Figure 1A–D) [11].

AMPA receptors are the primary members of the ionotropic glutamate receptor family that mediate fast excitatory synaptic transmission in the CNS [11,13]. Upon glutamate binding, a conformational change ensues the opening of AMPAR ion channels, allowing the influx of sodium ions (Na_+_) into the postsynaptic neuron, generating EPSCs [14]. These EPSCs are essential for initiating action potentials and propagating excitatory signals within neural circuits, which consequently leads to the activation of NMDA receptors [15]. Delta receptors, however, are different from the other members of the iGluR family in terms of their lack of typical glutamate-induced ion channel currents [16]. Both the subtypes of delta receptors (GluD1 and GluD2) are characterized by their unique ligand-binding domains (LBDs), contributing to their divergent electrophysiological and gating properties compared to AMPA, Kainate, and NMDA receptors [17]. This member of the iGluR family was named as an “orphan receptor” for many years due to the absence of identified endogenous interacting ligands. However, recent studies have shed light on the interaction of GluD subunits with glycine and D-serine in vitro and situ (Figure 1D) [12,18]. The roles of these receptors in synaptic plasticity are particularly prominent, with GluD2 being essential for cerebellar long-term depression (LTD) and proper cerebellar function, and GluD1 implicated in synapse formation and maintenance in regions such as the prefrontal cortex and hippocampus [19,20,21]. Collectively, ionotropic glutamate receptors play a crucial role in the healthy functioning of the mammalian central nervous system. Dysfunction of the iGluRs is associated with a variety of neurological disorders, including autism spectrum disorder, epilepsy, Alzheimer’s disease, schizophrenia, and Huntington’s disease [22,23,24,25].

According to the Genome Aggregation Database (gnomAD), approximately 700 variants of the GRIN protein family have been identified in humans. Notably, GRIN2 protein subunits account for the majority of these variants, which include frameshift, nonsense, missense, splice-site, deletion, inversion, and translocation mutations associated with neurodevelopmental disorders (NDDs) [26]. Positioning these variants within GRIN2 subunits is critical for determining both NMDAR physiology and the phenotypes linked to NDDs. For instance, individuals with missense variants in the TMD + Linker domain of GRIN2A subunits present with more severe developmental delay (DD) and intellectual disability (ID) phenotypes compared to those with variants in the ATD + LBD regions [27]. Similar findings have been reported regarding seizure types in epilepsy patients, where epileptic spasms are exclusively observed in individuals with missense variants in the TMD + Linker regions of GRIN2A subunits [27]. Additionally, residues within the M3 transmembrane helix (M3-TMH) are highly conserved among various GRIN subunits and contain the SYTANLAAF motif, which is broadly consistent throughout the glutamate receptor family. NMDARs containing variants in the M3-TMH have been identified in patients with epilepsy, DD, ID, and autism spectrum disorder (ASD). These variants are associated with altered agonist potency, modified sensitivity to endogenous inhibitors, changes in divalent ion permeability, disrupted receptor surface trafficking, and altered electrophysiological properties [28].

Understanding the pathophysiology of ionotropic glutamate receptors is essential for clarifying their roles in NDDs and developing targeted therapeutic approaches. Various disease models, including Drosophila, mouse, non-human primate, zebrafish, and human-derived induced pluripotent stem cells (hiPSCs), have been established to explore the pathophysiology of NMDARs in NDDs [29,30,31,32,33]. These models provide critical insights into the molecular mechanisms that underlie these disorders by allowing researchers to manipulate specific genes and assess their effects on brain development and function. Moreover, they facilitate the identification of risk factors and potential therapeutic targets while serving as essential platforms for preclinical drug testing. This review centers on exploring the structural aspects, functional mechanisms, and pathophysiological implications, focusing on the NMDARs, and disease models pertinent to characterizing NMDA receptors in NDDs.

## 2. Structure and Function of NMDA Receptors

Characteristic structural features that are conserved among different subtypes of iGluRs include the extracellular amino-terminal domain (ATD) which contains an agonist-binding site, ligand-binding domain (LBD) that is proximal to the plasma membrane, a transmembrane domain (TMD) for enabling ion permeation into the cells, gating elements that control the opening and closing of the permeation pore, and an intracellular C-terminal domain (CTD) for downstream signaling [11]. NMDA receptors (NMDARs) are cation-specific ligand-gated ion channels that mediate glutamatergic synaptic transmission throughout the CNS. The NMDARs are localized on presynaptic, postsynaptic, and extrasynaptic membranes and play pivotal roles in excitatory neurotransmission and synaptic plasticity [34,35]. Functional NMDARs generally exhibit a heterotetrameric structure that is formed by combination of GluN1 (NR1), GluN2A-D (NR2A-D), and GluN3A-B (NR3A-B) subunits [36]. Notably, GluN2 subunits show distinct temporal and spatial expression patterns which play a critical role in neural development. For instance, GluN2B is predominantly expressed in early developmental stages and contributes to synaptic plasticity and connectivity during brain maturation. As development progresses, there is a shift toward GluN2A expression, which is associated with refined synaptic function and more stable connectivity in mature neural circuits. Alterations in the expression patterns of GluN2 subunits are linked to neurodevelopmental disorders, where atypical temporal or spatial expression disrupts the balance of excitatory and inhibitory signaling, potentially contributing to conditions like autism and schizophrenia [37].

**Figure 2:**
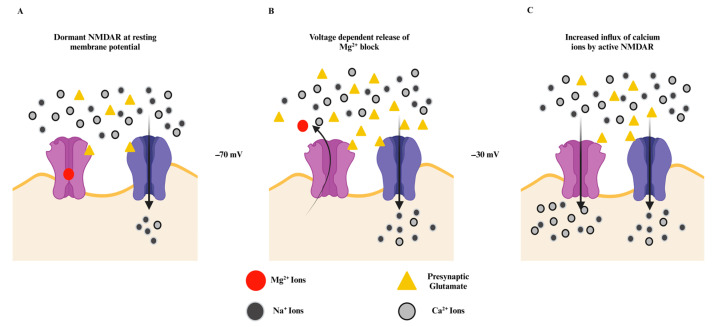
Activation of the “coincidence receptor”. (**A**) Initial interaction of AMPAR (blue) and NMDAR (Purple) with pre-synaptic glutamate; the receptors are inactive at the resting membrane potential (−70 mV); upon interaction with the presynaptic glutamate, NMDAR remains inactive still since Mg^2+^ is blocking the ion channel pore, while AMPA receptor allows in-flow of Na^+^ ions initiating the depolarization of the postsynaptic membrane. (**B**) Depolarization of the postsynaptic membrane facilitates the release of Mg^2+^ ions from the NMAR ion channel pore, activating the receptor. (**C**) Activation of NMDAR and repetitive presynaptic glutamate release leads to increased in-flow of Ca^2+^ and Na^+^ ions into the postsynaptic neurons. The representation is adapted from Sprengel et al. 2022 [43]. This fundamental characteristic of NMDA receptors is disrupted in NDDs, where disease-associated variants are distributed across various domains of the GRIN proteins within NMDARs, impacting multiple physiological properties and leading to either receptor hypofunction or hyperfunction. Missense variants within the transmembrane helix may alter NMDAR surface expression and modify receptor sensitivity to endogenous agonists and inhibitors [2]. Likewise, variants in the extracellular ATD and LBD regions are often linked to receptor loss of function in DD/ID patients [27]. These alterations impact the neurons’ downstream calcium signaling, which affects long-term potentiation and synaptic plasticity in NDD patients.

The initial depolarization of NMDA receptors is stimulated by its interaction with the presynaptically released glutamate and extracellularly abundant glycine, followed by an influx of Na^+^ ions, generating the EPSCs [11]. Another temporally distinct component contributing to these EPSCs arises from the APMA receptors. The AMPA receptors mediate a synaptic current with rapid rise and decay kinetics; NMDA receptors, on the other hand, exhibit relatively slow kinetics with an activation time of approximately ten milliseconds (rise time) and deactivation time (decay time) in the range of tens to thousands of milliseconds [38]. This difference in the kinetics enables depolarization of NMDAR and voltage-dependent release of Mg^2+^ ions. The slow synaptic currents enable prolonged influx of Ca^2+^ ions into the postsynaptic neurons (Figure 2). This substantial increase in Ca^2+^ concentration stimulates intracellular signaling, which modulates gene regulation to induce various processes and behaviors in postsynaptic neurons, including synaptic plasticity [39], maintenance of dendrite arborization for memory formation [40], neuroprotection [41], and regulation of synapse formation [42].

### 2.1. Glycine-Binding GluN1 Subunit

**Figure 3:**
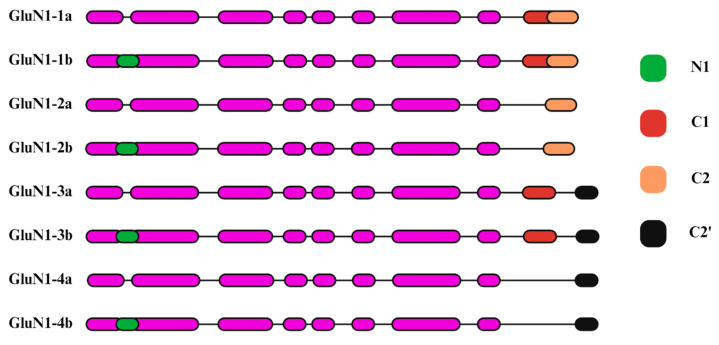
Schematics of the eight functional isoforms of the GluN1 subunit resulting from alternative RNA splicing. Color coding represents the splicing sites at exons 5, 21, and 22 or 22’, denoted as cassettes N1, C1, C2, and C2’, respectively. N1 cassette site is in the amino-terminal domain, whereas C1, C2, and C2’ sites are in the C-terminal domain. The schematics are adapted from Li et al., PNAS, 2021 [45].

In the CNS, the GRIN1 gene encodes the GluN1 subunits of the NMDA receptor, which harbor the glycine-binding sites and act as a fundamental component of the functional receptors. GluN1 subunits exhibit constitutive expression during developmental stages and are notably prevalent across various brain regions compared to GluN2 subunits of the NMDA receptors [44]. The GRIN1 gene consists of 22 exons, of which three exons undergo alternative RNA splicing and generate eight functional isoforms of the GluN1 protein (Figure 3) [45]. The GluN1 variants are referred to as N1, C1, C2, and C2’. These polypeptide cassettes are encoded through different splicing combinations of exons 5, 21, and 22 or 22’. Exons 5 and 21 encode for 21 amino acids (N1) in the ATD and 37 amino acids (C1) in the CTD, respectively. The C2 cassette within the CTD, encoded by Exon 22, comprises 38 amino acids. The deletion or splicing out of the C2 segment leads to the elimination of the initial termination signal, leading to a modified open reading frame (ORF) that integrates alternative 22-amino-acid cassette (denoted as C2’) into the C-terminal of the final protein product [46]. The CTD of GluN1 subunits interact with various intracellular proteins, such as postsynaptic density protein 95 (PSD95), which regulates the trafficking and localization of NMDA receptors [47]. Additionally, GluN1 CTD interaction with calmodulin and neurofilaments plays a role in activating regulatory proteins (ex. CaMKII) and maintaining the structural integrity of neuronal axons, respectively [48,49].

The splice variants of GRIN1 can form homomeric ion channels that exhibit ligand-gated ion-channel properties, like the NMDA receptors. However, the homomeric receptors differ in physiological and pharmacological properties, such as their affinity towards exogenous and endogenous agonists, polyamine potentiation, and protein kinase C regulation [50,51]. Apart from the homomeric receptors, the identity of GluN1 variants in the di-heteromeric and tri-heteromeric receptors also plays a crucial role in defining the kinetic and pharmacological properties of NMDARs. Exon 5 undergoes alternative splicing, giving rise to two distinct isoforms of the GluN1 protein, GluN1-1a and GluN1-1b. GluN1-1a is distinguished by excluding residues encoded by exon 5, whereas GluN1-1b contains the peptide sequence encoded by exon 5 [46]. Rumbaugh et al. have shown that the deactivation kinetics of GluN2B subunits are influenced by specific residues encoded by exon 5 within the GluN1 subunit. Specifically, GluN2B receptors deactivate more rapidly when paired with GluN1-1b [52]. Similar observations were made by Vance et al. in Xenopus oocytes, where GluN1 splice variants affected the agonist potency, deactivation time course, and single channel properties of GluN2D containing NMDA receptors [53]. This study examined the effect of exon-5 encoded residues of the GRIN1 subunit on the overall functionality of GluN1-GluN2D containing NMDA receptors. Including exon-5 encoded residues resulted in prolonged deactivation and reduced interaction with agonists L-glutamate and glycine [53]. Moreover, agonist stereochemistry also acts as a contributing factor in regulating receptor deactivation time course. For instance, linear agonists such as D-glutamate, L-aspartate, and D-aspartate resulted in faster deactivation of Glun1/Glun2D containing NMDARs compared to L-glutamate [54]. GluN1-1a plays a crucial role in long-term potentiation, with experimental data indicating that the synaptic potentiation in GluN1-1a mice is significantly greater than in GluN1-1b mice. Accordingly, GluN1-1a knockout mice carrying a deletion of the exon 5 of Grin1 demonstrate markedly improved spatial memory acquisition and recall [55]. N-terminal splicing of GluN1 is crucial for excitatory synapse maturation and neuronal network stability. Deletion of Grin1 exon 5 disrupts NMDAR development, leading to an overproduction of excitatory synapses in cortical layer 5 pyramidal neurons and increased seizure susceptibility in adult mice [56], highlighting the significant role of GluN1 splicing in regulating synaptic dynamics and network excitability.

### 2.2. The GluN2 Diversity and Its Role in the NMDA Receptors

The incorporation of two GluN1 subunits is indispensable for the constitution of operational NMDAR complexes. However, the assortment of GluN2/3 subunits governs the receptor’s functional versatility. In contrast to the GluN1 subunit, GluN2 expression fluctuates dynamically during embryonic brain development, reflecting an intricate regulation of stage-specific, region-specific, and cell-specific expression patterns [57]. During embryogenesis in rodents, a widespread distribution characterizes both GluN2B and GluN2D subunits. As the developmental period progresses, GluN2B maintains a high level of expression restricted to the forebrain, while GluN2D diminishes notably, primarily localizing to midbrain structures like the diencephalon and mesencephalon in adulthood [58,59]. Conversely, GluN2A emerges at birth, progressively amplifying its presence across the CNS, and is particularly enriched in regions of high cognitive function, such as the cortex and hippocampus [59,60]. GluN2C-containing receptors emerge during the first postnatal week, maintaining a localized expression within the olfactory bulb, thalamus, and vestibular nuclei. Eventually, during the second week, a developmental transition from GluN2B- to GluN2C-containing NMDARs is observed in cerebellar granule cells, which remains the primary site of GluN2C expression in adult rats [44,59,61]. The intricacies of GluN2 subunit distribution in the mammalian brain further entail the cell-specific expression of GluN2-containing NMDARs. For instance, despite their low expression in the cortex and hippocampus, GluN2C and GluN2D exhibit selective expression in interneurons and glial cells [62,63]. Similarly, GluN2B and GluN2D localize in cerebellar Golgi cells despite their overall low expression in the cerebellum [64,65]. Within the mature forebrain, GluN2A-containing receptors are predominantly localized at the synaptic terminals. In contrast, GluN2B receptors exhibit a broader spatial distribution, spanning peri- or extra-synaptic regions. Remarkably, synaptic NMDAR subunit composition displays dynamic adaptability in response to neuronal activity and environmental stimuli, affording precise modulation of receptor subtypes during synaptic plasticity [66,67].

The temporospatial distribution of the GluN2 proteins is regulated by alternative splicing, which is a pivotal mechanism influencing the expression patterns of GluN2 proteins, shaping their functional diversity and regulatory dynamics within the CNS. Based on the human-brain RNA-seq data, a Glun2A isoform (GluN2A-short) was identified, which was characterized by the excision of 343 nucleotides from the final canonical exon of GRIN2A gene, thereby yielding a C-terminally truncated GluN2A with 1281 amino acids (aa) [68]. Warming et al. demonstrated that the GluN2A-short subunit is abundantly expressed (up to 25%) in the human brain and forms functional receptors upon co-expression with the GluN1 subunit [69]. Its prevalence extends to chimpanzee and macaque datasets but is notably absent in rats and mice, indicating its primate-specific nature [68,70,71]. The truncation of the GluN2A CTD bears significant functional implications, notably resulting in the loss of critical interaction motifs that act as the binding sites for other regulatory proteins, such as CaMKII and PSD-95 [57]. A previous study by Tabish et al., investigating GluN2B transcript variants in a mouse model, reported two transcripts of the NR2B gene arising from alternate splicing in the noncoding exons of the 5′ untranslated region (UTR) [72]. Interestingly, GluN2C, predominantly expressed in the cerebellum, exhibits alternative 5′-UTR exons, particularly GRIN2C-a, which appears more abundant than the canonical exon 1, suggesting a primary UTR isoform. Additionally, a less common isoform (GRIN2C-b) with an alternative splice site at the 5′-exon junction was observed along with the elongation of exon 2, indicating variability in the 5′-UTR of the GRIN2C gene [68]. However, the RNA-seq data suggest no splice site consensus sequences are observed in rodents or primates for the GRIN2D gene [68,73]. These findings underscore the complexity of alternative splicing mechanisms and their implications for the diversity of GluN2 subunits in NMDA receptors. Understanding this intricate regulation is crucial for elucidating the roles of NMDARs in synaptic plasticity, neural function, and neurodevelopmental processes.

Translating findings from rodent models to human conditions requires caution due to the species-specific differences in brain structure and developmental timelines [74,75]. Advances in genetic engineering, such as humanized mouse models expressing human-specific NMDAR subunits, enhance translatability and allow the targeted exploration of GluN2-related therapeutic intervention [76]. Integrating rodent models with human-derived neural cultures and computational approaches may provide the most robust pathway for applying insights on GluN2 and NMDAR functions to clinical settings [77].

## 3. NMDA Receptors in Neurodevelopmental Disorders: Variants of GluN2 Subunits

The GluN2 subunits of NMDA receptors play pivotal roles in brain function and are critically involved in various neurological disorders. Each GluN2 subtype—GluN2A, GluN2B, GluN2C, and GluN2D—contributes to synaptic transmission, plasticity, and excitability, and their diverse combinations and interactions with other receptor subunits may influence pathological mechanisms underlying conditions such as epilepsy, ASD, DD/ID, schizophrenia, and other NDDs [25,78,79,80,81,82]. Identifying GluN2 variants associated with these disorders plays a crucial role in understanding the intricate dynamics of NMDAR pathophysiology and is essential for developing effective therapeutic strategies. The genome aggregation database (gnomAD) comprises over 700 variants of the GRIN genes that exhibit no correlation with the healthy population, out of which GRIN2A accounts for 44% of all known disease-associated variants of the GRIN family [26]. Disease-relevant variants are scattered across the domains of the mature protein, where mutations in the transmembrane domain and linker regions are known to be particularly associated with more severe phenotypes and poor prognosis [27,83].

The most common phenotypes associated with GRIN2A mutations are epilepsy/seizures, ID, and schizophrenia (SCZ) [84,85]. GRIN2A variants exhibit truncation and missense mutations in schizophrenia, resulting in haploinsufficiency and loss of function. The SCZ-linked GRIN2A variants predominantly result in loss of function in NMDARs. Meanwhile, M653I and S809R mutations linked to epilepsy and DD/ID may lead to gain-of-function or loss-of-function of NMDA receptors, depending upon the location of the mutation [86]. For example, in a SCZ cohort, a patient was observed to bear a de novo missense mutation (c.2902G>A; p.A968T) in the intracellular region of GRIN2A which did not have significant implications on NMDAR function [87]. Individuals with a severe developmental disorder and intellectual disabilities are more prone to the missense mutations in GRIN2A rather than the protein-truncation variants [88]. Given the diversity of subunit composition in NMDA receptors, GRIN2B might compensate for the loss of GRIN2A. However, electrophysiological data from the rats suggest that Grin2a+/− and Grin2a−/− knockouts exhibit reduced NMDA-evoked currents. Furthermore, percentage inhibition of NMDA-evoked currents in Grin2a−/− neurons after ifenprodil treatment is significantly greater than that of Grin2a+/− or Grin2a+/+ neurons, suggesting that subunit compensation does not occur in cases of allelic deletions [27,89]. Another study explored the impact of GluN2B variants (G689C and G689S) on NMDA receptor function across different receptor configurations (di- and tri-heteromers). These variants drastically reduced glutamate potency but exhibited partial restoration in mixed receptors (GluN2A and GluN2B) due to positive cooperativity. The neurosteroid Pregnenolone Sulphate was observed to effectively potentiate the receptors, indicating the therapeutic potential for GRINopathies despite concerns about non-specific effects [90].

GRIN2B variants account for around 37% of all variants observed in NMDA receptors in NDDs [26]. GRIN2B variants arising from missense, nonsense, frameshift, or splice site mutations are frequently associated with NDDs such as autism, schizophrenia, DD/ID, and epileptic encephalopathy. Mutations affecting the NMDAR function are distributed throughout the structural domains of the GRIN2B subunit [91]. Whole-exome sequencing data from patient cohorts suggest that rare de novo mutations in the ABD and TMD are observed exclusively in the patient population and significantly alter NMADR functional properties [78,79,92]. For example, gene screening and variation analysis on an ASD cohort identified a missense mutation (c.2473T>G; p.L825 V) in a highly conserved region of the transmembrane region of GRIN2B which was predicted to be relatively damaging in an in-silico pathogenicity analysis [87]. In another study, a trio-based exome sequencing of individuals with sporadic ASD was performed and 21 de novo mutations were identified, 11 of which were found to be protein-altering mutations. A single-base substitution was discovered in a proband at the canonical 3’ splice site of GRIN2B (exon 10), which resulted in possible regression and co-morbidity for mild ID [93]. Recently, it was established that the truncated GRIN2B resultant of the point mutation in the splice site mentioned above affects the cellular phenotype of neurons in ASD. The mutant subunit (GRIN2B724t), which is truncated in the second extracellular loop (S2), upon expression with the WT-GRIN1 subunit, hinders the trafficking of NMDAR to the cell surface or dendrites. Additionally, GRIN2B724t disrupts dendrite morphogenesis by decreasing outgrowth rates while increasing retraction and subsequent pruning [94,95].

While GRIN2-A/B variants are most frequently observed in the NDDs, GRIN2C and GRIN2D variants account for 2.8% and 3.8% of total GRIN variants identified in NMDARs, respectively [26]. The molecular characterization of postmortem brains from SCZ patients established a significant reduction in GRIN2C mRNA levels in the dorsolateral prefrontal cortex (DLPFC) tissue, which may lead to altered NMDAR stoichiometry and endogenous NMDAR deficit in schizophrenia [96,97,98,99]. Li et al. identified rare de novo GRIN2D variants in two independent probands with epileptic encephalopathy. A missense variant (c.1999G>A; p.Val667Ile) in the GRIN2D gene, resulting in a single amino acid substitution in the M3 transmembrane domain, was considered the most likely disease-causing variant [100]. Additionally, a GRIN2D mutation (c.1412G > A) potentially affects the splicing site, which may be a possible risk factor in SCZ [101].

## 4. Insights into NMDA Receptor Pathophysiology from Mouse Models of Schizophrenia and ASD

Rodents are extensively utilized in modeling human disorders due to several key factors. Their genetic similarity to humans provides a strong foundation for research. The ability to modify their genomes, combined with their rapid reproductive cycle, facilitates efficient study. Their small size and social behavior make them easy to maintain in laboratory settings. Researchers have developed a variety of neurological, behavioral, pharmacological, and other tests to assess features of neurodevelopmental disorders in these models, broadening our understanding of these conditions [102,103,104,105]. Additionally, mouse models have been instrumental in elucidating the underlying biological and pathophysiological mechanisms of NMDA receptor-related neurodevelopmental disorders [30,106,107,108]. For instance, mouse models with targeted genetic modifications in the NMDA receptor subunits to explore the complex neurobiology underlying schizophrenia (SCZ) have been previously developed that are instrumental in identifying abnormal neural circuits, synaptic dysfunctions, and behavioral abnormalities of the disorder [30,109,110]. Mouse models used to study schizophrenia often focus on NMDA receptor dysfunction. Over the years, various strategies, including pharmacological, genetic, and neurodevelopmental models, have been developed to study the NMDA receptor hypofunction in SCZ [111].

One of the approaches in pharmacological models entails the usage of NMDAR antagonists that act as open-channel blockers. Phencyclidine (PCP), ketamine, and MK-801, AKA the “trapping blockers,” get trapped inside the NMDAR ion channel pore, which renders the receptor in a closed inactive state [112,113,114,115]. MK-801 (dizocilpine) is a highly potent noncompetitive NMDA receptor antagonist, more effective than ketamine and PCP, and is, therefore, widely used in animal studies to induce schizophrenia-like behavioral and neurochemical changes [116,117]. Genetic analyses reveal that polymorphisms in the coding and promoter regions of GRIN subunits can affect NMDA receptor transcript levels and functionality. While various mouse models with targeted deletions of NMDA receptor subunits have been created, those with homozygous GRIN1 or GRIN2B subunits fail to survive past the perinatal period [118,119,120,121]. NR1 knockdown (KD) mice were designed to address lethality by expressing the NR1-1a splice variant in NR1 knockout mice. These NR1 KD mice exhibit altered dendritic differentiation, increased axonal arborizations, and faster development of corpus callosum projection neurons. However, their average lifespan depends on the transgene expression levels, limiting their use for further behavioral, molecular, and structural studies in adults [122,123]. Another NR1 KD mouse line, created by inserting a neomycin cassette into the GRIN1 gene, shows significantly reduced NR1 expression. Nonetheless, these mice exhibit a range of schizophrenia-like behaviors, including impaired social and sexual interactions, cognitive inflexibility, abnormal ERPs, spatial cognition, sensorimotor gating deficits, hyperlocomotion, stereotypy, self-injury, decreased anxiety, and reduced nest building [106,124,125,126,127,128].

Comparable strategies are employed in developing mouse models for ASD, using genetic modifications and behavioral assessments to investigate NMDA receptor dysfunction and its role in ASD. For instance, in 16p11+/− mice, which exhibit deficits in spatial memory and social motivation, NMDA receptor activation in the medial prefrontal cortex (mPFC) layer five pyramidal neurons is notably reduced. This impairment in NMDA receptor function is attributed to decreased phosphorylation of the GluN2B subunit at the S1303 site rather than a decrease in the overall expression of the subunit. To counter this, Wang et al. used a chemogenetic strategy involving the “designer receptors exclusively activated by designer drugs (DREADDs)” and delivered via viral vectors to the mPFC. The activation of these receptors was done with clozapine-N-oxide (CNO)-facilitated, CaMKII-mediated phosphorylation of the S1303 site on the GluN2B subunit. This led to the successful restoration of NMDA receptor-mediated EPSCs to normal levels and alleviated the behavioral deficits observed in the mice [129]. Interestingly, zinc, co-released with glutamate and accumulating in postsynaptic spines, significantly modulates NMDAR function. Zinc deficiency is a significant factor in the etiology of behavioral defects associated with ASDs [130,131], and studies have shown that dietary supplementation of zinc can help alleviate the phenotypes of ASD [132]. Dietary zinc supplementation significantly improved auditory fear memory and social interaction in Tbr1+/− mice [133]. In Grin2b mice with ASD-risk mutation (Grin2b+/C456Y), reduced GluN2B levels led to decreased hippocampal NMDAR currents, enhanced LTD, and normal LTP, suggesting protein degradation and LTD sensitivity. These mice had normal social behavior but showed increased anxiolytic-like behavior. Early D-cycloserine treatment rescued NMDAR function and improved adult anxiolytic behavior [134].

Other than NMDAR-associated ASDs, several other risk genes have been identified, out of which the SHANK family genes are particularly implicated in severe behavioral deficits [135,136]. SHANK variants are critical in determining the specific type of NMDA receptor functional deficits observed in ASD mouse models [137]. For example, mice with either haploinsufficiency (Shank3e4–9+/−) or complete deficiency (Shank3e4–9−/−) in the ankyrin repeat (ANK) domain of SHANK3 exhibited a significant decrease in the NMDA/AMPA excitatory postsynaptic current (EPSC) ratio at cortical excitatory synapses onto striatal medium spiny neurons. Contrastingly, in a Shank313–16−/− mice, which lacks the PSD95/DlgA/Zo-1 (PDZ) domain-coding exons 13–16 of Shank3, NMDAR-mediated synaptic transmission at striatal synapses remained normal; however, the decay kinetics of NMDAR-mediated EPSCs were altered [138,139].

Overall, mouse models serve as significant research tools for studying NMDAR-associated NDDs by providing a platform to investigate the effects of NMDA receptor dysregulation on brain function and behavior. Through targeted genetic modifications and detailed behavioral assessments, these models have been crucial in uncovering the underlying mechanisms and elucidating the complex interactions involved in these disorders. Despite their utility, mouse models of neurodevelopmental disorders have considerable limitations, as they do not fully replicate the complexity of human disease conditions and provide a limited understanding of the etiopathology of the disease. Moreover, special care is required in some instances, as the pharmacologic agents, such as PCP, used for inducing the disease phenotype can render mice hyperexcitable and aggressive [140,141,142]. Primate models overcome some limitations of rodent models, such as brain anatomy and drug response differences. Still, they only replicate basic behaviors like simple social interactions and repetitive actions [31,143], and stringent ethical limitations restrict their use. These limitations hinder the study of complex behavioral changes in NDDs and the development of effective therapies. This warrants an alternative model system that can replicate the phenotypes relevant to the human NDDs.

## 5. Human iPSC-Derived Neurons in the Research of NMDA Receptor Pathophysiology

**Figure 4:**
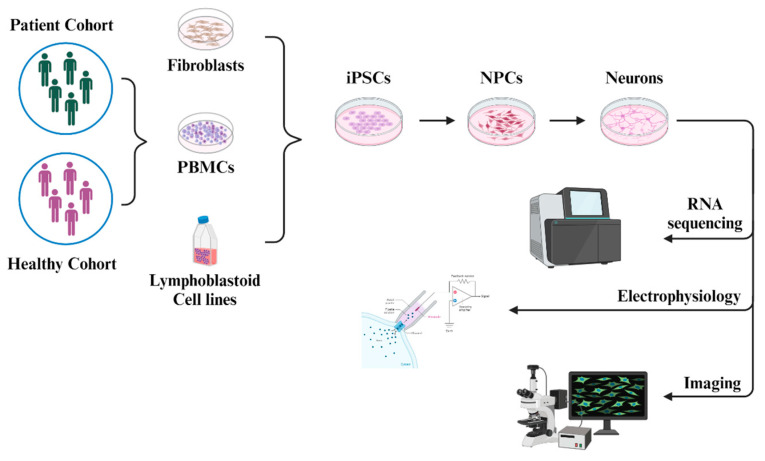
Modeling neurodevelopmental disorders using patient-derived iPSCs. The illustration depicts the general protocol for modelling human neurodevelopmental disorders (NDDs) using patient-derived induced pluripotent stem cells (iPSCs). The iPSCs can be derived from the fibroblasts, PBMCs, or lymphoblastoid cell lines (LCLs) of the patients as well as the healthy subjects (Isogenic controls). These iPSCs provide a platform to study the disease biology and explore novel therapeutic strategies through various techniques that can lead to the development of personalized and more effective treatment options for patients suffering with different NDDs. The schematics of the figure are adapted from Liu et al., Development, 2018 [148]. iPSC-derived neurons from ASD patients frequently exhibit reduced synaptic activity and altered excitatory/inhibitory signaling balance, both of which are critical to understanding synaptic dysfunctions characteristic of ASD [149,150,151,152]. With their ability to model complex synaptic processes, aberrant connectivity, and neurotransmitter imbalances, hiPSC-derived neurons serve as invaluable tools for dissecting the molecular mechanisms underlying neuropsychiatric disorders such as ASD, schizophrenia, bipolar disorder, and ID [153,154,155,156,157]. Integration-free methods for iPSC generation avoids genomic integration of vectors, thereby preserving genetic integrity and reducing tumorigenic risks [158]. Consequently, several groups have successfully adopted this method for modeling different neurological disorders. For instance, iPSCs were generated by reprogramming fibroblasts derived from a Phelan-McDermid syndrome (PMS) patient, harboring an insertion mutation in SHANK3 (C.3679insG) [159]. The iPSCs were observed to express the pluripotency markers, differentiate into the three germ layers, retain the disease-causing mutation, and display normal karyotypes. Therefore, this technology allows researchers to explore the functional properties of cellular factors involved in the pathology of NDDs, which can be translated into a patient specific therapeutic intervention.

The emergence of human induced pluripotent stem cell (hiPSC) technology stands as a pivotal milestone in biomedical research, offering a potent tool to explore human biology and diseases in unprecedented detail. Since its inception following Yamanaka’s groundbreaking work in 2006–2007, hiPSCs have provided researchers with an unparalleled opportunity to unlock the secrets of human development and pathophysiology in vitro [144,145]. By reprogramming somatic cells into a pluripotent state, hiPSCs enable the generation of patient-specific cellular models, circumventing ethical concerns and offering a unique window into the molecular underpinnings of various disorders [146]. The neurons from patient cells faithfully recapitulate critical aspects of human brain development and pathology. This allows researchers to explore the nuanced interactions between genetic predispositions, environmental factors, and neuronal phenotypes (Figure 4) [147].

In the CNS, the diversity among NMDAR subunits shapes receptor properties and functions, impacting both physiological and pathological conditions. GluN2 subunits demonstrate unique expression patterns across various brain regions and neuron types. Notably, GluN2A and GluN2B are prominently present in complex brain structures like the cortex, suggesting their pivotal roles in neural processes [11,58,160]. Expression of functional NMDA receptors as well as other ion channels in hiPSCs, which exhibit normal electrophysiological characteristics, serves as a bona-fide system for studying human brain in dish [161,162,163]. For instance, whole-cell patch-clamp experiments suggest that hiPSC-derived neurons have spontaneously occurring mEPSCs, indicating the presence of functional synapses in the cells [164]. Studies have shown mRNA expression of NMDAR subunits, such as GRIN1, GRIN2(A-D), and GRIN3(A-B), in neurons derived from human induced pluripotent stem cells (hiPSCs) [77,165]. Additionally, electrophysiology experiments indicate that while GluN2A subunits mediate 10–30% of NMDA-induced currents, GluN2B subunits account for over 70% in neurons differentiated via an NPC stage and NGN2 neurons, highlighting the predominance of functional GluN2B subunits in these cells [164]. The decay time constant of NMDAR-mediated synaptic currents provides further insight into the subunit composition of NMDARs in hiPSC-derived neurons. A prolonged decay time, exceeding 250 milliseconds, indicates that the synaptic currents are dominated by the GluN2b containing receptors [164,166]. Moreover, loss-of-function mutation in GRIN2B results in the impairment of differentiation and increased proliferation in hiPSC-derived neural progenitor cells (NPCs) [167]. The distinct expression patterns and functional roles of NMDAR subunits, especially GluN2A and GluN2B, highlight their significance in brain physiology. In line with this, researchers have utilized iPSC-derived neurons to study the molecular mechanisms underlying NDDs, including ASD, schizophrenia, and epilepsy.

### 5.1. iPSC Models for Studying NMDARs in ASD

Recent studies emphasize the effective use of iPSC-derived neurons to model ASD in the investigation of intricate neuronal phenotypes [150,168,169,170]. For example, a de novo heterozygous point mutation in Down Syndrome Cell Adhesion Molecule (DSCAM) gene was associated with ASD. iPSCs derived from a 12-year-old patient were used for generating neuronal cells. The DSCAM mutation resulted in the impairment of the NMDAR function, which was significantly restored by the exogenous expression of DSCAM [33]. Dysregulation of the SHANK family of proteins is also implicated in ASD. Located at the core of the postsynaptic density (PSD) in glutamatergic synapses, SHANK proteins bind to structural proteins, glutamate receptors, and the actin cytoskeleton, thereby modulating the structure, plasticity, and maturation of excitatory synapses [171,172]. SHANK3 plays an important role in regulating the functional properties of NMDA and AMPA receptors. It enhances synaptic transmission mediated by these receptors and boosts glutamate release by forming trans-synaptic signaling complexes with neurexin and neuroligin [173]. Recently, it was established that SHANK3 mutation is associated with S-nitrosylation (SNO), a nitric oxide-mediated posttranslational modification (PTM), which leads to neuronal pathology [174]. Consequently, the effect of SNO was studied parallelly in an ASD mouse model and hiPSC-derived neurons. The study demonstrated elevated levels of nitric oxide, which induced oxidative and nitrosative stress leading to the enrichment of SNO-proteome. Post neuronal nitric oxide synthase (nNOS)-inhibitor treatment, the ASD deficits were significantly restored in both the models [150].

Advanced techniques like transcriptome screening and whole-exome sequencing have expanded the list of ASD-associated genes and helped integrate these genes into relevant biological pathways, deepening our understanding of the underlying mechanisms [149,175]. Hussein et al. investigated the physiological properties of ASD-patient-derived neurons harboring mutations in different candidate genes including, SHANK3, UBTF, and GRIN2B. Interestingly, electrophysiological recordings of cortical neurons corresponding to all the mutants exhibited a similar phenotype of early maturation and hyperexcitability, suggesting common pathological pathways despite different genetic alterations [176]. Furthermore, hiPSCs provide a platform to decipher intrinsic differences in the pathology of NDDs among patients containing similar genetic makeup, indicating a role of environmental factors and epigenetic modifications in the manifestation of neuropsychiatric diseases [177,178]. These findings suggest that human-derived iPSC models can perfectly recapitulate the pathophysiological behaviors observed in ASD patients. Moreover, reprogrammed cells can be valuable for pinpointing shared pathway disruptions in the development of different NDDs such as ASD and SCZ. Studies on neurons derived from patients with ASD and SCZ using induced pluripotent stem cells (iPSCs) reveal distinct yet converging developmental patterns. Initially, neurons from ASD patients mature rapidly, showing increased excitability, enhanced sodium and potassium currents, and more extensive branching and synaptic connections compared to control neurons. However, as they continue to develop, these neurons lose their early advantages, becoming less excitable, with reduced synaptic activity and fewer branched neurites. In contrast, neurons derived from SCZ patients begin with lower functionality, characterized by less branching, reduced excitability, and decreased synaptic activity. Although SCZ neurons consistently develop more slowly than control neurons, they eventually exhibit a phenotype like mature ASD neurons, suggesting that while the developmental trajectories differ, both disorders result in comparable neurodevelopmental disruptions [179].

### 5.2. iPSC Models for Studying NMDARs in Schizophrenia

Schizophrenia is a complex psychiatric disorder with a global prevalence of about 1%, typically manifesting in late adolescence or early adulthood. SCZ is a highly heterogeneous disorder with variable clinical presentations, complicating the ability to draw generalized conclusions from postmortem data. For instance, postmortem studies on schizophrenia patients have reported inconsistent expression of the GRIN1 subunit in the frontal cortex, due to the differences in examined regions and variations in tissue handling and dissections [98,180,181,182]. Therefore, patient-derived iPSCs serve as a great alternative model system to overcome these limitations [183]. In a pioneering study, Chiang et al. derived integration-free iPSCs from schizophrenia patients with the DISC1 mutation, a genetic variant linked to the disorder’s pathology. These iPSCs displayed the characteristic morphology of human embryonic stem cells (hESCs), exhibited normal karyotypes, and expressed a range of pluripotency markers such as Nanog, Oct4, Sox2, SSEA3, SSEA4, TRA-1-60, TRA-1-81, and TRA-2-49 [184]. A subsequent in-depth study on iPSC-derived neurons from a schizophrenia patient revealed key functional deficits, including reduced neurite length and decreased levels of the synaptic protein PSD-95 [185]. Several studies have explored iPSC-derived neurons from schizophrenia patients, consistently finding abnormalities in neuronal and synaptic development, as well as in various intracellular signaling mechanisms [186,187,188,189,190]. Schizophrenia concordance rate in monozygotic twins ranges between 41–79%, which is much higher compared to that of dizygotic twins (10–19%) [191,192,193]. Despite the high concordance in monozygotic twins, the almost 50% of cases in these subjects exhibit discordance for SCZ [194]. The iPSC-derived neurons serve as a great model to study SCZ pathology in monozygotic twins, as it can measure disease associated changes in a similar genetic background. In line with this, a recent study has shown that iPSC-derived dentate gyrus (DG) neurons of affected individuals are less arborized and exhibit delayed maturation in comparison to the unaffected and control twins. Consequently, the excitatory postsynaptic currents are drastically reduced in the DG neurons of affected twins. Moreover, genetic alterations were observed in pathways related to neuronal development, synapse-related, and Wnt signaling in the affected individuals [177].

In iPSC-derived neurons from patients with schizophrenia, researchers have identified changes in the expression of various glutamate receptor subunits, including GRIN2A, GRIN2B, GRIK1, GRIK2, and GRM1, GRM7, as well as glutamate transporter genes [183]. The glutamate hypothesis, supported by findings that NMDA receptor antagonists can induce psychosis in healthy individuals, suggests that impairments in glutamatergic neurotransmission, involving NMDA receptors, play a crucial role in SCZ [195]. This hypothesis is further supported by reduced mismatch negativity (MMN), an event that indicates early dysfunctions in auditory processing and may signal the onset of schizophrenia-related alterations in brain function from a young age [196,197]. The PDE4 inhibitor rolipram was recently used to restore synaptic function and boost excitatory synaptic activity in iPSC-derived neurons with DISC1 mutations [198]. Additionally, PDE4 inhibitors have demonstrated clinical benefits in improving mismatch negativity (MMN) and correcting working memory-related theta activity changes [199,200]. Moreover, recent research highlights the involvement of ADCYAP1, the gene responsible for encoding the PACAP protein, in several disrupted pathways in neurons derived from schizophrenia patients [201]. PACAP plays a critical role in enhancing NMDA receptor function by activating the cAMP/PKA pathway, which results in the release of RACK1 from GRIN2B subunits [202]. A deficiency in PACAP, commonly seen in schizophrenia, likely reduces NMDA receptor activity, particularly in SST interneurons, which are highly enriched with PACAP receptors and GRIN2B subunits. This impairment may contribute to the underlying neural dysfunction associated with the disorder.

Numerous strategies have been devised for therapeutic targeting of NMDA receptors in SCZ. NMDARs are targeted in schizophrenia treatment to address symptoms that current antipsychotics fail to manage effectively, such as negative symptoms and cognitive dysfunction. Research focuses on various strategies to modulate NMDAR function. These include targeting the glycine modulatory site (GMS) on the NMDA receptors with compounds like GlyT-1 inhibitors [203], D-serine [204], DAAO inhibitors [205], and D-cycloserine (DCS) [206]. Additionally, positive allosteric modulators (PAMs) for metabotropic glutamate receptors, such as mGluR5 and Group II mGlu receptors, are explored to indirectly influence NMDAR function. Antioxidants like N-acetylcysteine (NAC) and sulforaphane are also investigated for their potential to mitigate NMDAR hypofunction. Despite mixed clinical outcomes—where some drugs show promise while others fail—this approach highlights the ongoing effort to refine therapeutic strategies and improve the efficacy of treatments targeting NMDAR dysfunction in SCZ [207].

### 5.3. iPSC Models for Studying NMDARs in Epilepsy

Induced pluripotent stem cell (iPSC) models have emerged as powerful tools in epilepsy research, offering unique opportunities to study the cellular and molecular mechanisms underlying the disorder. Patients with mutations in the same gene can exhibit widely varying seizure types, severities, and responses to treatment. iPSC models derived from these patients have been instrumental in studying several neurodevelopmental disorders associated with epilepsy. These include conditions such as Dravet Syndrome [208], Angelman Syndrome [209], CDKL5-related neurodevelopmental delay [210], STXBP1-associated epileptic encephalopathy [211], and Timothy Syndrome [212]. Rett syndrome is one of the most thoroughly investigated epileptic disorders using iPSC technology, caused by mutations in the MeCP2 gene on the X chromosome, with 50% to 90% of patients experiencing seizures [213]. iPSC models have shown reductions in neuronal soma size, neurite outgrowth, synapse formation, and spontaneous activity compared to controls [146,214]. Furthermore, wild-type neurons display similar morphological abnormalities when co-cultured with astrocytes from Rett syndrome patients, highlighting the significant role of astrocytes in epileptogenesis [215].

Recent research has highlighted the significant role of NMDA receptors (NMDARs) in the pathophysiology of epilepsy, particularly through genetic mutations and excitotoxic signaling pathways [216]. Mutations in genes encoding NMDAR subunits, such as GRIN1, GRIN2A, GRIN2B, GRIN2C, and GRIN2D, have been linked to various epileptic phenotypes, ranging from focal and generalized seizures to severe epileptic encephalopathies [216]. These mutations often disrupt NMDAR function, leading to increased neuronal excitability, which is central to epileptogenesis. For example, mutations in GRIN1 are associated with early infantile encephalopathy and developmental delay [217], while GRIN2A and GRIN2B mutations contribute to epilepsy syndromes like Landau–Kleffner syndrome and epileptic encephalopathy, respectively [218,219]. In addition to genetic factors, NMDAR-mediated excitotoxicity, triggered by excessive glutamate release, plays a pivotal role in neuronal death. This mechanism, characterized by calcium overload and activation of destructive cellular pathways, is not only relevant to epilepsy but also to neurodegenerative diseases like Alzheimer’s and Parkinson’s [216]. Targeting NMDAR dysfunction and excitotoxicity thus represents a promising therapeutic avenue for addressing refractory forms of epilepsy. In line with this, researchers have developed patient-derived iPSC models for studying NMDAR dysfunction in epilepsy. For instance, iPSCs were generated from peripheral blood mononuclear cells (PBMCs) from a seven-year-old child with epilepsy, who carried a heterozygous mutation of GRIN2A gene (c.1832 A>T) [220]. Another group generated iPSCs from fibroblasts of a developmental and epileptic encephalopathy (DEE) patient carrying a de novo heterozygous pathogenic variant of GRIN2D [221]. Similarly, Shi et al. developed an iPSC line from a patient with autosomal dominant neurodevelopmental disorder carrying the GRIN1 c.389A > G (p.Asp130Gly) mutation. This cell line serves as a valuable model for investigating GRIN1-related disorders, including epilepsy [222]. Overall, the integration of iPSC models in epilepsy research holds promise for enhancing our understanding of the disease’s heterogeneity and developing personalized therapeutic approaches for patients with various genetic backgrounds.

## 6. Discussion

This review delves into the multifaceted roles of NMDA receptors (NMDARs) within the central nervous system (CNS) and their critical involvement in various neurodevelopmental disorders. The intricate interplay of GluN2 subunits in shaping receptor functionality and synaptic plasticity underscores the essential contributions of these receptors to brain physiology. The literature suggests that the variability in NMDAR subunit composition is a fundamental factor influencing receptor kinetics and pharmacological properties. Particularly noteworthy are the differential expression patterns of GluN2 subunits across various developmental stages and brain regions, highlighting their distinct contributions to neurodevelopmental processes. The discovery of primate-specific isoforms, like the truncated GluN2A-short, adds another layer of complexity, suggesting unique evolutionary adaptations that may influence cognitive functions. The impact of NMDAR dysfunction becomes strikingly apparent when we consider the disease-associated GRIN gene variants linked to a spectrum of neurological disorders, including epilepsy, schizophrenia, and ASD. The prevalence of GRIN2A and GRIN2B variants in these conditions correlates with their critical roles in synaptic signaling. The complexity of NDDs is further compounded by the variable effects of different types of mutations on NMDA receptor function. For instance, while certain missense mutations may subtly alter receptor kinetics, others might drastically impair receptor function or localization [223,224]. Similarly, truncation mutations often lead to a complete loss of function, but their effects can vary depending on the specific region of the gene that is affected. Studies have shown that missense mutations in GRIN2A are frequently observed in patients with severe developmental disorders and intellectual disabilities, whereas protein-truncating variants tend to be less common. On the other hand, truncation mutations can have more pronounced effects, such as the GRIN2B truncation mutation that disrupts NMDAR trafficking and dendritic development, particularly in ASD [95,225]. This heterogeneity in mutation effects highlights the importance of developing sophisticated approaches to unraveling genotype–phenotype correlations.

Translating NMDAR-targeted therapies from preclinical models to clinical applications has encountered numerous challenges, largely due to the complexity of NMDA receptor function and the diversity of subunit compositions involved in different brain processes. While therapeutic agents like memantine and amantadine have demonstrated clinical utility, their broader application is limited by side effects [226]. Selective modulators such as ifenprodil, a GluN2B allosteric modulator, have shown promise with fewer adverse effects, but their therapeutic use remains constrained by poor bioavailability and limited receptor selectivity. These compounds often interact with other neurotransmitter receptors, further complicating their clinical utility [227]. Newer agents like fluoroethylnormemantine (FNM) and esmethadone (REL-1017), which are currently in clinical trials for neurodegenerative disorders, show potential due to their rapid therapeutic effects and reduced side effect profiles. However, their long-term efficacy and safety still require thorough validation in clinical settings [228]. Many NMDAR-targeted therapies that demonstrated efficacy in preclinical studies have struggled to replicate these results in human trials, often due to unanticipated side effects or an incomplete understanding of NMDAR function in human neurophysiology. For example, D-cycloserine, originally developed for tuberculosis, showed promising results in animal models of schizophrenia by improving cognitive function and memory, but clinical outcomes have been inconsistent and its use has been linked to neurotoxic side effects, including anxiety, depression, and seizures [229]. Advances in drug delivery systems present potential breakthroughs for overcoming these challenges. Adeno-associated viruses (AAVs), for instance, are promising vectors for targeted gene delivery [230]; however, their use for large genes, such as GRIN2B, remains problematic due to size constraints. Lipid nanoparticles (LNPs), on the other hand, are being developed to target neurons specifically, though crossing the blood-brain barrier (BBB) remains a significant challenge [231]. Exosomes, which can pass the BBB [232], offer another promising delivery mechanism [233], although they have yet to receive FDA approval for clinical use [234]. Additionally, CRISPR technology holds transformative potential for precise genetic interventions, yet delivering it effectively to brain tissues is still challenging [234]. The future of NMDAR-targeted therapy may lie in developing multifunctional agents that not only modulate NMDA receptors but also address other pathological processes, such as oxidative stress and neuroinflammation. Advances in drug delivery systems, including nanoparticle formulations, could also improve the bioavailability and therapeutic efficacy of these treatments. Ultimately, a deeper understanding of NMDAR physiology in humans and more refined therapeutic strategies are critical for advancing the clinical success of NMDAR-targeted therapies for neurodevelopmental and neurodegenerative disorders. The use of mouse models and patient-derived induced pluripotent stem cell (iPSC) neurons has significantly enriched our understanding of NMDAR-related pathophysiology. These models offer a window into the functional consequences of GRIN mutations in a controlled environment. Mouse models, engineered to carry human-relevant mutations, provide vital insights into the in vivo effects of NMDAR dysfunction. For instance, mouse models of autism spectrum disorders (ASD) with genetic modifications have shed light on specific types of NMDA receptor functional deficits. In 16p11+/− mice, which exhibit deficits in spatial memory and social motivation, NMDA receptor activation in the medial prefrontal cortex (mPFC) layer 5 pyramidal neurons is notably reduced due to decreased phosphorylation of the GluN2B subunit at the S1303 site; similarly, the Shank3e4–9+/− and Shank3e4–9−/− mice, which have haploinsufficiency or complete deficiency in the SHANK3 gene, show a significant decrease in the NMDA/AMPA excitatory postsynaptic current (EPSC) ratio at cortical excitatory synapses onto striatal medium spiny neurons. These findings suggest that mouse models are essential for understanding the pathophysiology of NMDA receptors in neurodevelopmental disorders by elucidating the specific genetic and molecular mechanisms regulating receptor function. Despite their utility, mouse models of neurodevelopmental disorders have limitations; they do not fully replicate the complexity of human disease conditions and they provide a limited understanding of the etiopathology of the disease. This limitation hinders the study of complex behavioral changes in NDDs and the development of effective therapies. Complementing the insights gained from animal models, patient-derived iPSC neurons offer a human-specific perspective. The emergence of human-induced pluripotent stem cell (hiPSC) technology stands as a pivotal milestone in biomedical research, offering a potent tool to explore human biology and diseases in unprecedented detail. Neurons derived from patients faithfully recapitulate key aspects of human brain development and pathology, allowing researchers to explore the nuanced interactions between genetic predispositions, environmental factors, and neuronal phenotypes. Importantly, findings from iPSC-derived neurons can also be directly correlated with clinical observations in patients with specific GRIN mutations, providing insights into the phenotypic expression of these mutations. For instance, studies utilizing iPSC-derived neurons from patients with mutations in neurodevelopmental genes such as SHANK3 and NRXN1 have demonstrated alterations in synaptic function and excitability that align with clinical manifestations, such as hyperexcitability and synaptic transmission deficits, seen in patients [235,236]. These correlations between in vitro neuronal phenotypes and patient symptoms underscore the translational relevance of iPSC models for understanding GRIN-related pathologies, enabling researchers to explore the functional consequences of NMDAR subunit mutations. Such correlations not only validate iPSC findings but also support their potential for identifying biomarkers and tailoring therapeutic strategies based on patient-specific cellular phenotypes. The study of GRIN-related pathologies enhances our understanding of the phenotypic spectrum associated with NMDAR-subunit variants. However, both animal models and iPSC-derived neurons present methodological limitations that must be acknowledged. Animal models, while invaluable, often fall short in replicating the full complexity of human synaptic architecture and circuitry [237,238]. This limitation restricts the ability to extrapolate findings directly to human pathophysiology, particularly in the study of synaptic interactions unique to the human brain Additionally, there are multiple types of mutations in the NMDA receptors and there is a need to develop a mouse model for each specific mutation. Similarly, iPSC-derived neurons, although human-based, are subject to variability in their responses, stemming from differences in patient genetic backgrounds, reprogramming techniques, and differentiation protocols [239]. Furthermore, iPSC-derived models may not fully capture the three-dimensional organization and synaptic connectivity seen in the intact human brain, which are essential for accurately modeling complex neurodevelopmental and neuropsychiatric conditions [240]. Addressing these limitations through advancements in model refinement and validation remains crucial for developing a comprehensive understanding of NMDAR-related pathologies and improving translational relevance.

For instance, some GRIN2B variants are linked to NDDs like ID, autism, and epilepsy. Functional analyses classify these variants as gain- or loss-of-function based on assays that assess key receptor properties such as agonist sensitivity and ion channel function. This framework for categorizing variants plays a critical role in precision medicine, helping to guide targeted therapies by determining whether a variant enhances or impairs NMDAR function, ultimately aiding in better diagnosis and treatment strategies for ion channel-related disorders [27,241]. Notably, NMDA receptors are critical for neurobehavioral functions, and their subunit expression is influenced by various environmental and pathological factors. One such factor is arsenic exposure, which has been shown to reduce NR2A mRNA levels in the hippocampus in a dose-dependent manner. In rats exposed to arsenic, NR2A expression decreased by 20–29% compared to controls, illustrating arsenic’s neurotoxic potential and its impact on NMDAR function [242]. This reduction in NR2A may impair cognitive processes associated with the hippocampus, emphasizing the importance of environmental toxins in modulating NMDAR activity and contributing to neurodevelopmental dysfunction. In contrast to the negative effects of toxins and injury, environmental enrichment (EE) positively regulates NMDAR subunit expression. In neonatal rodents treated with MK-801, a drug that downregulates NR1 subunit expression in the hippocampus, EE restores NR1 levels and promotes synaptic plasticity [243]. Additionally, EE also increases the expression of NR2A and NR2B subunits that are essential for learning and memory processes. Mice overexpressing NR2B exhibit enhanced cognitive abilities, while NR1 inactivation in the hippocampal CA1 region impairs memory [244]. Additionally, EE restores PSD-95, a postsynaptic protein that regulates NMDAR activity, further promoting synaptic plasticity and cognitive function [243].

Looking ahead, several avenues for future research stand out. There is a pressing need for more detailed studies on the less-explored GluN2C and GluN2D subunits, whose distinct expression profiles suggest unique functions in the CNS. While these subunits are less abundant than GluN2A and GluN2B, they appear to play specialized roles in regions associated with specific neurodevelopmental and neuropsychiatric disorders. A deeper understanding of GluN2C and GluN2D functions could yield targeted therapeutic approaches for conditions where these subunits are implicated. Additionally, the role of alternative splicing in generating NMDAR diversity warrants further investigation, particularly in the context of disease. Understanding these mechanisms could unlock new therapeutic targets, offering hope for more effective interventions. As we continue to advance our genomic and neuropharmacological tools, the potential for precision medicine in treating NMDAR-related disorders becomes increasingly tangible, promising a future where therapies are as unique as the patients they are designed to help. Techniques like CRISPR/Cas9 gene editing allow researchers to precisely manipulate NMDAR-related genes, enabling in-depth studies of individual subunits and mutations. Combined with patient-derived iPSC models and organoid technology, these approaches allow for more accurate disease modeling and drug screening which could identify compounds that selectively modulate specific NMDAR subunits with reduced side effects. Additionally, advances in 3D culture systems and brain organoids offer promising avenues to better mimic human neural circuitry, bridging the gap between basic research and clinical application. Emerging delivery methods, including exosomes, LNPs, and AAVs, further enhance the potential for precise, targeted interventions. As these technological and methodological tools continue to evolve, the potential for precision medicine in treating NMDAR-related disorders becomes increasingly tangible. This progress envisions a future where therapies are tailored to the genetic and molecular profiles of individual patients, ultimately leading to more effective and personalized interventions for NMDAR-linked neurodevelopmental disorders.

In conclusion, NMDARs are pivotal to both normal brain function and the pathogenesis of a wide range of neurodevelopmental disorders. By unraveling the complex genetic, structural, and functional aspects of these receptors, we can better understand their roles in health and disease, ultimately guiding the development of innovative therapeutic strategies. The ongoing progress in this field holds great promise for improving clinical outcomes and enhancing the quality of life for individuals affected by these challenging conditions.
